# Linking cortical microtubule attachment and exocytosis

**DOI:** 10.12688/f1000research.10729.1

**Published:** 2017-04-12

**Authors:** Ivar Noordstra, Anna Akhmanova

**Affiliations:** 1 Cell Biology, Department of Biology, Faculty of Science, Utrecht University, Padualaan 8, 3584 CH Utrecht, Netherlands

**Keywords:** exocytosis, cytoskeleton, mictrotubules, tracking proteins, cargo transport, IQGAP1

## Abstract

Exocytosis is a fundamental cellular process whereby secreted molecules are packaged into vesicles that move along cytoskeletal filaments and fuse with the plasma membrane. To function optimally, cells are strongly dependent on precisely controlled delivery of exocytotic cargo. In mammalian cells, microtubules serve as major tracks for vesicle transport by motor proteins, and thus microtubule organization is important for targeted delivery of secretory carriers. Over the years, multiple microtubule-associated and cortical proteins have been discovered that facilitate the interaction between the microtubule plus ends and the cell cortex. In this review, we focus on mammalian protein complexes that have been shown to participate in both cortical microtubule capture and exocytosis, thereby regulating the spatial organization of secretion. These complexes include microtubule plus-end tracking proteins, scaffolding factors, actin-binding proteins, and components of vesicle docking machinery, which together allow efficient coordination of cargo transport and release.

## Introduction

Exocytosis is a secretory trafficking process during which molecules are processed and transported to the cell surface, where they can be either released into the extracellular space or inserted into the plasma membrane. Secretory transport occurs in multiple steps: after budding from the Golgi, exocytotic vesicles travel along cytoskeletal filaments toward the cell periphery, come into contact with tethering factors that can restrain them, and subsequently dock and fuse with the plasma membrane with the aid of soluble NSF attachment protein receptors (SNAREs). Secretion can occur constitutively, to maintain cell homeostasis and provide components of extracellular matrix and cell adhesion structures (constitutive exocytosis). Alternatively, release of specific cargos in many types of differentiated cells can be tightly controlled in both space and time by a variety of signaling pathways (regulated exocytosis). Regulated exocytosis plays an important role in multiple processes, including synaptic neurotransmission, endocrine and paracrine signaling, or the release of hydrolytic enzymes by intestinal cells and leukocytes (for review, see 1, 2).

Vesicular transport is facilitated by the cytoskeleton, and in mammalian cells the major tracks for vesicle transport are microtubules, dynamic hollow tube-like structures with an outer diameter of 25 nm and lengths in the order of tens of microns. Microtubules have intrinsic polarity, with fast growing plus ends and slowly growing minus ends. Vesicles are transported along microtubules by two types of motors: kinesins, which are mostly plus-end-directed, and cytoplasmic dynein, which moves to microtubule minus ends.

In order to function optimally, cells rely heavily on a precisely controlled delivery of cargo. To do so, they take advantage of protein complexes that specifically connect membrane trafficking and cytoskeletal organization at the cell cortex. Tethering of microtubule tips, the end points of vesicle transport, to the sites of vesicle fusion can provide efficient routes for secretion. In many types of mammalian cells, microtubule minus ends are clustered at the internally positioned microtubule-organizing centers, the centrosome and the Golgi apparatus (for review, see 3– 5), and the secretory trafficking mainly takes place in the direction of microtubule plus ends. It should be noted that in polarized epithelia and in neurons, microtubule minus ends can also be positioned in the vicinity of cell cortex and serve as sites of vesicle delivery. Since the mechanisms responsible for cortical microtubule minus-end tethering are only beginning to be understood ^6– 11^, their connections to exocytosis still need to be unraveled.

In contrast, the factors responsible for coordinating the organization of microtubule plus ends and secretion have received much attention. For example, in different types of migrating cells, secretory traffic is preferentially directed toward the leading cell edge ^12, 13^. Such polarization of exocytosis can help to establish and maintain cell asymmetry and provide molecules needed for membrane protrusion. An important function of exocytosis is formation and modification of cell adhesions to extracellular matrix or other cells. In particular, it is well established that microtubule plus ends can be specifically linked to the vicinity of focal adhesions (FAs) to promote their remodeling and thus facilitate efficient cell movement (for review, see 14, 15).

Complexes responsible for coordinating microtubule plus-end organization and exocytosis consist of molecules localized to microtubule plus ends and cortical proteins, which can participate, often through additional factors, in vesicle tethering and docking. At the cortex, these complexes typically comprise different scaffolds associated with the actin cytoskeleton or directly with the plasma membrane. On microtubules, the major players are microtubule plus-end tracking proteins (+TIPs), a heterogeneous class of proteins distinguished by their specific accumulation at the growing microtubule plus ends (for review, see 16, 17). Here, we provide an overview of the mammalian +TIPs involved in cortical microtubule tethering, their associated cortical attachment complexes, and their roles in exocytosis.

## +TIPs involved in cortical microtubule capture

Prominent factors that can autonomously recognize growing microtubule ends are the members of end binding (EB) protein family ^18– 20^. EBs recruit to microtubule tips a plethora of different binding partners, which fall into two major classes: proteins containing globular cytoskeleton-associated protein-glycine-rich (CAP-Gly) domains and proteins with a short linear motif Ser-any amino acid-Ile-Pro (SxIP) embedded in unstructured positively charged regions (for review, see 17). Mammalian +TIPs well known for their involvement in cortical microtubule capture are the CAP-Gly-containing cytoplasmic linker protein of 170 kDa (CLIP-170), p150Glued, the large subunit of the dynein co-factor dynactin, the SxIP proteins CLIP-associating proteins CLASP1/2 and the tumor suppressor adenomatous polyposis coli (APC).

**Figure 1.**
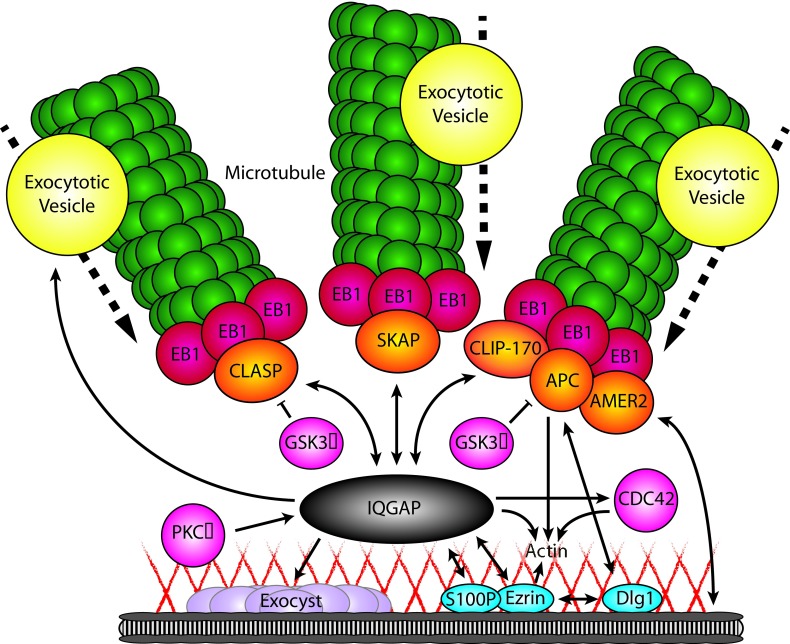
Schematic overview of IQGAP and associated protein functions in cortical microtubule capturing and exocytosis. Through its different domains, IQGAP interacts with a subset of microtubule plus-end tracking proteins (+TIPs) (orange) and cortical proteins (blue), thereby facilitating the microtubule capture at sites with high exocytotic activity. At the same time, IQGAP controls multiple components involved in secretion, including exocytotic vesicle-specific Rab GTPases (yellow), the exocyst complex (purple), and actin (red lines). Single-headed arrow, protein interaction with regulatory function; double-headed arrow, protein interaction facilitating recruitment; bar-headed line, negative regulation; dotted arrow, movement in direction of arrow.

CLIP-170, the first +TIP to be reported ^21, 22^, was proposed to be involved in tethering microtubules to the cell cortex via IQGAP1 ^23^, a cortical scaffold protein with interesting roles in exocytosis which we will discuss below ( Figure 1). In fibroblasts, IQGAP1 recruits CLIP-170-decorated microtubule plus ends to actin filaments at the leading edge during migration ^24^. Interestingly, subsequent biochemical studies showed that IQGAP1 appears to act as a cortical hub for multiple +TIPs: for example, it can interact with APC, which is found in the same protein complex as CLIP-170 ^24^ ( Figure 1). Upon the depletion of APC, the leading edge localization of IQGAP1 as well as CLIP170 was perturbed and directional migration was affected, suggesting that APC, CLIP170, and IQGAP act in a tripartite complex that mediates cortical anchoring of microtubules during cell movement ^24^. Immunoprecipitation experiments from fibroblasts also revealed an interaction between IQGAP1 and CLASP2, which was implicated in polarized cell movement ^25^ ( Figure 1). Furthermore, a complex of IQGAP1 with SKAP, an SxIP protein originally identified as a +TIP linking kinetochores to spindle microtubules ^26, 27^, was shown to orchestrate directional migration by coupling dynamic microtubule plus ends to cortical regions in breast cancer cells ^28^ ( Figure 1). It should be noted that the evidence for the function of IQGAP1 as a cortical hub for different +TIPs strongly relies on protein interaction data and would profit from additional mechanistic cell biological analyses.

APC, which was shown to directly bind to EB1 ^29^, has been implicated in multiple additional cortical microtubule stabilization pathways. *In vivo*, it localizes to actin-rich cortical protrusions where it directly interacts with actin filaments through its C-terminal basic domain ^30, 31^. On the basis of *in vitro* experiments, APC was also suggested to play a role in actin nucleation ^31^. Furthermore, APC was shown to stabilize microtubules at the cortex in migrating fibroblasts by acting together with the actin-nucleating factor of the formin family, mDia ^32^. In migrating astrocytes, APC directly interacts with the cortical scaffold protein Dlg1 ^33^, thereby directly linking microtubules to the cortex. The latter interaction is regulated by the kinase GSK3β, which in turn can be phosphorylated by a Par6-PKCζ complex ^33^.

**Figure 2.**
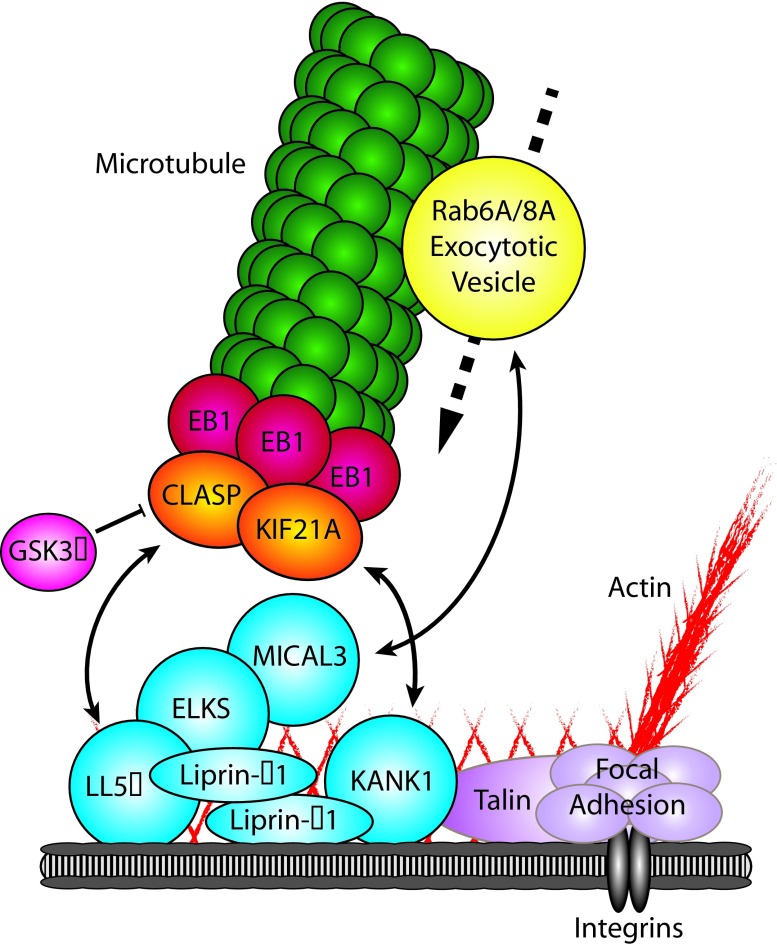
Schematic overview of the cortical microtubule stabilizing complex (CMSC) and associated proteins involved in microtubule capture and exocytosis. The CMSC (blue) captures microtubules through direct interactions with a specific subset of microtubule plus-end tracking proteins (+TIPs) (orange). CMSC components are found in the proximity of focal adhesions (purple), where they regulate microtubule-mediated focal adhesion turnover. Furthermore, the CMSC has been shown to directly interact with Rab6A/Rab8A-positive vesicles (yellow), thereby facilitating secretion. Double-headed arrow, protein interaction facilitating recruitment; bar-headed line, negative regulation; dotted arrow, movement in direction of arrow.

CLASP1 and CLASP2 form another family of major microtubule regulators that accumulate at the microtubule plus ends at the front of migrating cells. The asymmetric CLASP distribution is mediated by their spatially controlled phosphorylation through GSK3β, which reduces their affinity for microtubule plus ends ^25, 34, 35^. CLASPs are recruited to the cell cortex by directly interacting with the phosphatidylinositol (3,4,5)-trisphosphate (PIP3)-binding protein LL5β ^36^ ( Figure 2). LL5β is part of a large protein assembly tightly linked to FAs, which controls FA turnover (see below). Another SxIP-containing +TIP shown to participate in organizing microtubules in the vicinity of FAs is the APC-binding protein AMER2/FAM123, which is directly linked to the plasma membrane by a phospholipid-binding domain ^37– 39^ ( Figure 1).

Also, the dynein-dynactin complex represents an important player in microtubule capture at the cortex. In contrast to other +TIPs, which promote lateral microtubule attachments to the cortical sites, cytoplasmic dynein can form end-on attachments and exert forces to position the whole microtubule network in both interphase and mitosis ^40^ (for review, see 41, 42). Though mostly studied during cell division, when secretion is downregulated, cortical dynein was also shown to play a role in microtubule tethering to the plasma membrane in neurons, where it acts together with the neural cell adhesion molecule, and may have a role in stabilization of synapses ^43^.

Finally, spectraplakins, a group of very large proteins, have been shown to be involved in cortical microtubule stabilization. Spectraplakins have the ability to directly link microtubules and actin filaments and are involved in a wide range of cellular processes. Despite the presence of only two mammalian genes, a variety of spectraplakin isoforms have been found. This results from the existence of alternative tissue-specific promotors and multiple splice variants (for review, see 44). Among them, actin crosslinking factor 7 (ACF7), also known as microtubule actin crosslinking factor 1 (MACF1), has been studied most extensively. Owing to the intrinsic properties of a C-terminal microtubule-binding domain and the presence of an SxIP domain, it can interact with microtubules and specifically accumulate at their ends. At the same time, the N-terminal calponin homology domains mediate the binding to sites rich in actin, such as the cell cortex ^45^. Depletion of ACF7 was associated with the impaired microtubule growth along F-actin fibers toward FAs, and this significantly affected FA turnover and cell migration ^46^. Cortical recruitment of ACF7 has been connected to the presence of membrane-bound APC, which in turn is regulated by GSK3 ^47^, indicating the interplay of multiple pathways in recruiting microtubules to FAs. Recently, ACF7 was also shown to be a key player in linking microtubule minus ends to the apical membrane of polarized epithelial layers through calmodulin-regulated spectrin-associated protein 3 (CAMSAP3) ^7, 8, 10^. Interestingly the ACF7-CAMSAP3 interaction was also associated with FA targeting and cell migration ^9^.

Among the numerous links between microtubules and the cell cortex, two broad groups of protein assemblies with clear connections to secretion have emerged—IQGAP-containing complexes and the CLASP- and LL5-containing cortical microtubule stabilization complexes—and these will be discussed in more detail below.

## Coordination of cytoskeletal cortical interactions and secretion by IQGAP1

As mentioned above, IQGAP1 interacts with multiple +TIPs, thereby facilitating the capture of microtubules at specific cortical cell regions. At the same time, IQGAP1 plays a role at different steps of the secretory pathway, ranging from actin remodeling to the control of specific membrane trafficking regulators, such as Rab GTPases or the exocyst complex. This functional diversity is based on the presence of multiple domains, including a calponin homology domain, IQGAP-specific repeats, a calmodulin-binding motif, a RasGAP-related domain, and a RasGAP C-terminus, which can mediate binding to a surprisingly broad set of proteins.

IQGAP1 is linked to the cortex via S100P and the plasma membrane- and actin-binding protein ezrin ^48, 49^ ( Figure 1). S100 proteins bind to Ca ^2+^ and the interaction between S100P and IQGAP1 is strictly Ca ^2+^-dependent ^48^. Also, ezrin has been shown to bind to Ca ^2+^-bound S100P and IQGAP1, but since ezrin and IQGAP1 do interact in the absence of Ca ^2+^, this interaction appeared to be S100P-independent ^49^. Both S100P and ezrin co-localize with IQGAP1 in the cortical cell regions, and ezrin depletion reduced the cortical localization of IQGAP1 ^48, 49^. Interestingly, ezrin also interacts with the APC-binding protein Dlg1 ^50^, but it is not known whether APC, Dlg1, IQGAP1, and ezrin can function in the same complex.

IQGAPs are best known as important regulators of actin dynamics. In turn, the actin cytoskeleton plays a major role in regulating all steps of exocytosis. Multiple studies show that the actin network acts as a physical barrier that is removed during exocytosis, allowing vesicles to dock and fuse with the plasma membrane (for review, see 51, 52). Many lines of research also indicate the role of actin in directing vesicles to the fusion sites, regulating the fusion pores and providing the driving force to complete fusion ^53– 55^. Undoubtedly, actin regulation is essential for properly functioning exocytotic machinery.

IQGAP1 was initially identified as a target for the Rho GTPases CDC42 and Rac1 ^56, 57^, two factors involved in actin organization. Despite the name, IQGAP1 displays no GAP activity to the Rho GTPases ^56– 59^. In fact, it is well established that IQGAP1 inhibits the GTPase activity of CDC42 and Rac1 to stabilize their GTP-bound form ^58, 60, 61^. Accumulated evidence points in the direction of CDC42 being an important regulator of post-Golgi traffic in an actin-dependent manner ^62, 63^. Interestingly, the CDC42-IQGAP interaction was directly linked to exocytosis in gastric parietal cells, epithelial cells that are located in the gastric glands of the stomach. In these cells, IQGAP1 and its homologue IQGAP2 are expressed and localized differentially ^64, 65^. In contrast to IQGAP1, which localizes to the basolateral regions of the cells, IQGAP2 specifically localizes to the apical plasma membrane, where it interacts with CDC42. This interaction was shown to be essential for polarized secretion ^64^. Biochemical evidence demonstrated that IQGAP1 can be phosphorylated by the kinase PKCε at its C-terminus, thereby relieving an autoinhibited fold, enhancing the binding of IQGAP1 to active CDC42 ^66^, and leading to attenuation of exocytosis ^67^. PKCε has also been implicated in exocytosis by playing an essential role in the disassembly of actin filaments following docking and tethering of the vesicles ^68– 70^. Since different stages of exocytosis require different actin organizations, these data suggest a dynamic interplay between PKCε, CDC42, and IQGAP in regulating actin dynamics.

In addition to interacting with indirect actin modifiers like the Rho GTPases, IQGAP1 also binds to a set of proteins that directly organize the actin cytoskeleton, such as the actin-related protein (Arp) 2/3 complex and formins. IQGAP can stimulate Arp2/3-dependent actin polymerization through direct as well as indirect interactions via the activation of neural Wiskott-Aldrich syndrome protein (N-WASp) ^71, 72^. Also, mDia1, an actin-nucleating protein of the formin family, which was implicated in microtubule regulation through APC and other pathways ^32, 73, 74^, was identified as a binding partner for IQGAP1. IQGAP1 specifically interacts with the Rho-activated form of mDia1 which results in the recruitment of the protein and actin assembly at sites with high exocytotic activity, like the leading edge of migrating cells ^75, 76^.

Next to Rho GTPases, Rab GTPases also belong to the key regulators of membrane trafficking and exocytosis. Interestingly, Rab27A, a small GTPase that regulates exocytosis of insulin-containing vesicles in pancreatic β cells ^77^, has been shown to form a complex with IQGAP1 ^78^. Remarkably, not only exocytosis but also endocytosis of insulin secretory membranes, a process essential to maintain a constant cell volume and to allow the reuse of exocytotic machinery, strongly depends on complex formation between Rab27A and IQGAP1. Depletion of IQGAP1 prevented glucose-induced redistribution of Rab27A from the cytosol to the plasma membrane ^78^. These data suggest that IQGAP1 participates in both endocytosis and exocytosis upon glucose stimulation in β cells. Whether these functions relate in some way to the interactions of IQGAP1 with microtubule-binding proteins is currently unclear, especially as, strikingly, microtubules in β cells restrict, rather than promote, the availability of insulin granules for secretion ^79^.

IQGAP1 has been shown to associate with the Exo70, Sec3, and Sec8 subunits of the exocyst complex ^67, 80^ ( Figure 1), an evolutionarily conserved octameric protein complex, which mediates the tethering of exocytotic vesicles prior to fusion and which is implicated in a wide variety of cellular processes (for review, see 81). The IQGAP1-exocyst interactions are controlled by CDC42 and RhoA ^67, 80^. Interestingly, depletion of IQGAP1 strongly affected insulin secretion from pancreatic β cells ^67^ and secretion of matrix metalloproteinases ^80^, two unrelated cellular processes which both strongly rely on exocytosis and the exocyst complex ^82, 83^. However, it cannot be excluded that these phenotypes are caused by other functions of IQGAP1 in exocytosis as described above.

Taken together, the existing data suggest that IQGAP1 is an excellent candidate for playing the role of a central hub coordinating cytoskeletal organization and membrane trafficking. However, more detailed biochemical and cell biological studies will be needed to understand the exact mechanisms underlying its activity and unravel which of the numerous proposed interactions and functions of IQGAP1 are compatible and cooperative and which ones are mutually exclusive.

## CLASP- and LL5-associated complexes in microtubule organization and secretion

As mentioned above, CLASPs are among the key players responsible for cortical microtubule targeting. Through the direct interaction with LL5β and its homologue LL5α ^36, 84^, they associate with a large protein assembly, which here will be termed cortical microtubule stabilizing complex (CMSC) ^36, 85– 87^ ( Figure 2). As discussed below, this complex has been shown to be a regulator of FA turnover and is tightly clustered at the rims of FAs, although it does not spatially overlap with them ^36, 85– 88^. LL5s are PIP3-binding proteins, and their membrane recruitment as well as the localization of the whole CMSC can be influenced by PI3 kinase activity ^36, 89^. Recently, Prickle1, a protein known for its role in planar cell polarity, was shown to participate in the LL5β-dependent accumulation of CLASPs in close proximity to FAs at retracting cell edges, thus controlling FA disassembly and cell motility ^90^.

CMSC contains several scaffolding proteins, including the SAM domain containing proteins liprin-α1 and -β1, a coiled coil adaptor ELKS (also known as ERC1, for ELKS/RAB6-interacting/CAST family member 1), and the ankyrin repeat protein KANK1. Liprin-α1 and -β1 were initially identified as interacting partners of the protein tyrosine phosphatase LAR ^91^, a transmembrane protein that is involved in axon guidance (for review, see 92) and in the maintenance of excitatory synapses in hippocampal neurons ^93^. However, it is unknown whether LAR homologues are present and have a functional role in CMSCs. Liprin-α1 directly interacts with ELKS ^94^. Both ELKS and the members of the liprin-α family are major components of the cytomatrix at the active zone (CAZ), the principal site of Ca ^2+^-dependent exocytosis of neurotransmitters at neuronal synapses; these proteins thus have complex roles in neurotransmission across different animal species (for review, see 95– 97). Importantly, CMSC and CAZ have many non-overlapping components; for example, CAZ does not contain either LL5 or KANK homologues and does not appear to be directly connected to microtubules. In addition to coordinating the trafficking of neurotransmitter-containing vesicles at the CAZ, liprin-α1 was shown to be a key component of the molecular machinery underlying the internalization of fibronectin and recycling of fibronectin-bound α5β1-integrin to basolateral membranes in endothelial cells, a process essential for defining and maintaining cell polarity ^98^.

CMSCs are linked to FAs by KANK1, which directly interacts with talin, the core FA protein ^87^ ( Figure 2). KANK1 also binds to liprin-β1, and the inhibition of either the liprin-β1-KANK1 or the KANK1-talin binding disrupts the CMSC localization around FAs and cortical microtubule capture around FAs ^87^. How a direct interaction between an FA component and a CMSC component can lead to their non-overlapping localization next to each other is currently a mystery. KANK2, a KANK1 homologue, also localizes as a tight “belt” around FAs and interacts with talin ^99^. Interestingly, it suppresses mechanical force transmission across activated integrins by interfering with F-actin binding ^99^. The fact that talin might interact with KANK1 and actin in a mutually exclusive manner could explain why KANKs localize to the periphery of FAs and not to their actin-associated core. It is possible that KANK1 bound to the FA periphery acts as a “seed” for CMSC assembly through multivalent interactions between its scaffolding components ^87^ (for review, see 100). In addition to coupling the CMSCs to FAs, KANK1 recruits to the cortex the kinesin-4 family member KIF21A. This plus-end-directed motor protein strongly inhibits both microtubule growth and catastrophes at the cell cortex, thus cooperating with CLASPs in promoting cortical microtubule stability ^85^.

ELKS is a ubiquitously expressed adaptor, which can be recruited to the plasma membrane by both LL5s and liprin-α1 ^36, 94^. The effect of ELKS depletion on the microtubule organization is relatively mild because ELKS does not bind to microtubules and is not essential for the cortical localization of LL5β or CLASPs but rather plays a scaffolding role by concentrating cortical clusters of LL5β and CLASPs at the cell periphery ^36^. However, ELKS has been shown to be a central player in constitutive exocytosis ^101^. It directly interacts with all isoforms of the small GTPase Rab6 (Rab6A, Rab6A′, and Rab6B) ^102^, an abundantly expressed Rab GTPase that strongly decorates the Golgi apparatus and cytoplasmic vesicles ^103– 105^. Although these vesicles were originally believed to be responsible for COPI-independent transport to the Golgi ^106, 107^, detailed imaging studies demonstrated that in fact they predominantly fuse with the plasma membrane and thus represent carriers of constitutive secretion ^101^.

Rab6A-positive vesicles immobilize and fuse at the cortical FA-associated sites containing LL5β, and ELKS depletion causes strong accumulation of Rab6A-positive vesicles at the cell periphery because although their exit from the Golgi and microtubule-based transport are not perturbed, their docking and fusion are inhibited ^101^. The underlying mechanism is not entirely clear. Similar to CAZ components, ELKS-containing complexes might promote the interaction between SNAREs located on the vesicles and the plasma membrane; however, it is currently unclear whether there is a direct connection between SNAREs and ELKS. Furthermore, in addition to the direct binding to ELKS, Rab6 also controls the recruitment to exocytotic vesicles of another Rab GTPase, Rab8A. Rab8A is a well-known player in exocytosis ^108– 111^. Interestingly, Rab8A interacts with ELKS-positive cortical sites through the binding partner of ELKS, MICAL3 ^112^, a multidomain oxidative enzyme which can promote disassembly of actin filaments and potentially remodel other protein complexes and also act as a scaffold ^113, 114^ (for review, see 115).

In migrating cells, CMSCs are strongly clustered around the FAs at the leading cell edge and promote their disassembly ^36, 116^. Microtubules anchored by CLASPs in the vicinity of FAs serve as tracks for transport of exocytotic Rab6-positive vesicles. Secretory trafficking delivers to the cell surface membrane type 1 metalloprotease (MT1-MMP), which can degrade the extracellular matrix around FAs, resulting in integrin detachment, loss of tension, and FA turnover ^116^. These observations help to explain why liprin-α1, liprin-β1, LL5β, and ELKS promote invasive behavior and internalization of integrins in breast cancer cells ^86, 88, 117– 119^. Importantly, MT1-MMP delivery and integrin recycling also strongly depend on endosomal trafficking, which requires microtubules (for review, see 120, 121). How exactly endosome trafficking connects to CMSC components deserves further investigation. Finally, it should be noted that in three-dimensional matrix invasion assays, the major function of cortical microtubule stabilization by CLASPs and possibly also their partners might be not only to direct vesicle traffic but also to mechanically support long protrusions that mesenchymal cells extend in three dimensions to penetrate between the matrix fibers ^122^.

LL5β and ELKS were also shown to concentrate at podosomes, actin-rich dynamic structures which can remodel the extracellular matrix ^123^; and CLASPs, together with a plus-end-directed kinesin-3 KIF1C, were shown to regulate podosome formation ^124^. Interestingly, podosome-like structures (“synaptic podosomes”) are also formed at neuromuscular junctions (NMJs) undergoing remodeling during postnatal stages of development, and LL5β, which strongly localizes to regions of high density of acetylcholine receptors at the NMJ, has been implicated in this process ^123, 125, 126^. At the NMJ, the complexes of LL5β and CLASPs were shown to capture microtubule plus ends and in this way create a route for the delivery of vesicles containing acetylcholine receptors to the postsynaptic membrane ^127– 129^. It is currently unknown whether ELKS participates in the regulation of the fusion of acetylcholine receptor-containing carriers with the plasma membrane, but this possibility seems quite likely, given the involvement of ELKS in secretion and the observation that ELKS is present at the NMJ ^130^. Taken together, these data show that CMSCs or complexes related to them in composition regulate both microtubule organization and secretion in different types of undifferentiated as well as differentiated cells.

## Conclusions and future directions

Microtubules play an essential role in exocytosis by serving as tracks for motor proteins that transport secretory carriers. The best studied mammalian cell models so far include migrating mesenchymal cells, in which a surprisingly diverse set of molecules is responsible for attaching and stabilizing microtubules to cortical sites close to the leading cell edge. An important unresolved question is whether the different complexes described so far, such as CMSCs and IQGAP-linked cortical assemblies, represent cooperating or redundant pathways or whether *in vivo* they act in the same or different cell and tissue settings. Addressing this question will require systematic analysis of all major players using the same cellular models and also exploring their expression and interactions in tissues. Relevant in this respect is the analysis of tissue-specific isoforms of the investigated proteins. For example, whereas IQGAP1 was extensively studied, much less is known about its homologues IQGAP2, which is enriched in the liver and stomach, and IQGAP3, which is mainly found in brain and lung tissue ^131^. Their domain composition is highly similar to that of IQGAP1 and given their specific expression in tissues with high exocytotic activity, they are interesting candidates for having profound but undiscovered roles in exocytosis.

Another interesting set of questions concerns the involvement of the discussed complexes in regulated secretion. There are strong data showing that many of the factors described above are important for regulated exocytosis. For example, ELKS and liprin-α are well-known players in neurotransmitter release in neurons and also are required for exocytotic release of inflammatory mediators by mast cells upon induction of allergic responses ^132^. ELKS was also found to coincide with the docking and fusion sites of insulin in a pancreatic β-cell line; consistent with this observation, ELKS clusters show significant overlap with the clusters of the SNARE syntaxin 1, and ELKS depletion strongly affected insulin exocytosis ^133^. However, as indicated above, the connections between CMSC components and SNAREs require further elucidation. Furthermore, microtubule plus ends do not appear to be directly connected to the sites of exocytosis in neurons or β cells. It is possible that microtubules are linked to secretory sites when relatively rapid transport of newly synthesized proteins from the Golgi apparatus is functionally important. Direct microtubule-based delivery might not be essential when an excess of secretory cargo is available or when extensive local recycling of secreted molecules takes place, as is the case in neurons. In some cell types, such as pancreatic β cells, microtubules may even be used to sequester rather than deliver secretory cargo ^79^. Investigating the diversity of the mechanisms responsible for vesicle delivery and fusion represents an exciting subject for future research.
